# 2-(Hydrazonomethyl)phenol

**DOI:** 10.1107/S1600536809045504

**Published:** 2009-11-07

**Authors:** Yan-Fang Shang, Qing-Ming Wang, Miao-Li Zhu, Yue-Hua Zhang

**Affiliations:** a School of Chemistry and Chemical Engineering, Nantong University, Nantong, JiangSu 226000, People’s Republic of China; b Institute of Molecular Science, Key Laboratory of Chemical Biology and Molecular, Engineering of the Education Ministry, Shanxi University, Taiyuan, Shanxi 030006, People’s Republic of China

## Abstract

The conformation of the title compound, C_7_H_8_N_2_O, is stabilized by an intra­molecular O—H⋯N hydrogen bond. The crystal structure shows inter­molecular N—H⋯O hydrogen bonds.

## Related literature

For Schiff bases as mixed-donor ligands in coordination chemistry, see: Lee *et al.* (2005 ▶). For the pharmaceutical and medicinal activity of Schiff bases, see: Sriram *et al.* (2006 ▶); Hao (2009 ▶); Bedia *et al.* (2006 ▶).
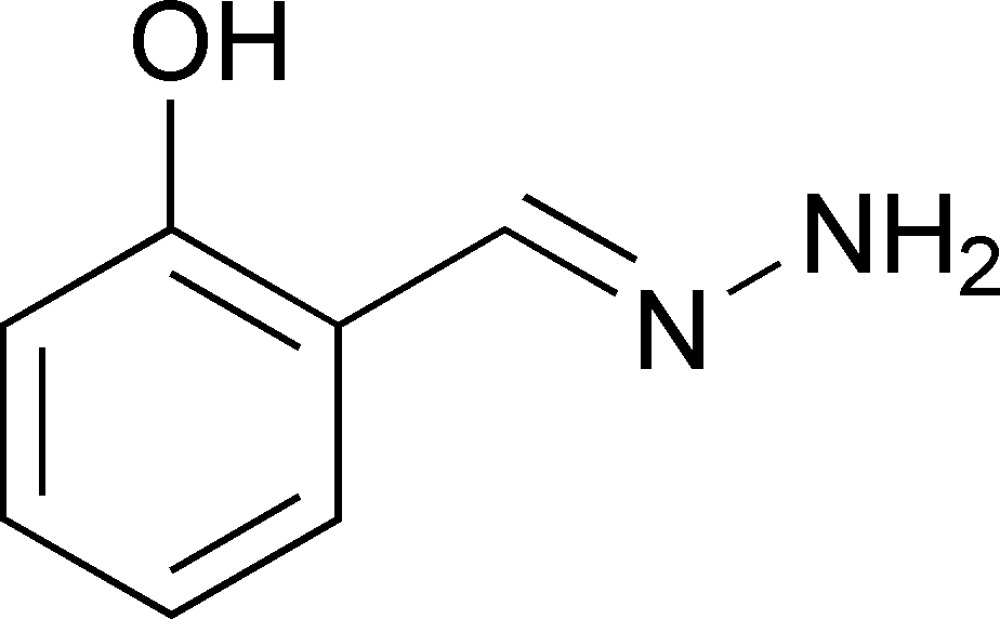


## Experimental

### 

#### Crystal data

C_7_H_8_N_2_O
*M* *_r_* = 136.15
Monoclinic, 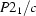

*a* = 14.1010 (11) Å
*b* = 6.0062 (5) Å
*c* = 8.1979 (6) Å
β = 102.5250 (10)°
*V* = 677.78 (9) Å^3^
*Z* = 4
Mo *K*α radiation
μ = 0.09 mm^−1^
*T* = 296 K
0.46 × 0.45 × 0.35 mm

#### Data collection

Bruker SMART CCD area-detector diffractometer
Absorption correction: multi-scan (*SADABS*; Bruker, 2000 ▶) *T*_min_ = 0.959, *T*_max_ = 0.968
3351 measured reflections
1203 independent reflections
1081 reflections with *I* > 2σ(*I*)
*R*_int_ = 0.013

#### Refinement

*R*[*F* ^2^ > 2σ(*F* ^2^)] = 0.036
*wR*(*F* ^2^) = 0.110
*S* = 1.06
1203 reflections
93 parameters
.
Δρ_max_ = 0.33 e Å^−3^
Δρ_min_ = −0.29 e Å^−3^

### 

Data collection: *SMART* (Bruker, 2000 ▶); cell refinement: *SAINT* (Bruker, 2000 ▶); data reduction: *SAINT*; program(s) used to solve structure: *SHELXS97* (Sheldrick, 2008 ▶); program(s) used to refine structure: *SHELXL97* (Sheldrick, 2008 ▶); molecular graphics: *XP* (Sheldrick, 2008 ▶); software used to prepare material for publication: *publCIF* (Westrip, 2009 ▶).

## Supplementary Material

Crystal structure: contains datablocks I, global. DOI: 10.1107/S1600536809045504/bt5112sup1.cif

Structure factors: contains datablocks I. DOI: 10.1107/S1600536809045504/bt5112Isup2.hkl

Additional supplementary materials:  crystallographic information; 3D view; checkCIF report

## Figures and Tables

**Table 1:** Hydrogen-bond geometry (Å, °)

*D*—H⋯*A*	*D*—H	H⋯*A*	*D*⋯*A*	*D*—H⋯*A*
N2—H2*A*⋯O1^i^	0.86	2.56	3.3076 (17)	145
N2—H2*B*⋯O1^ii^	0.86	2.23	3.0530 (16)	160
O1—H1⋯N1	0.82	1.89	2.6109 (15)	147

Symmetry codes: (i) 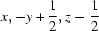; (ii) 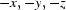.

**Fig. 1.**
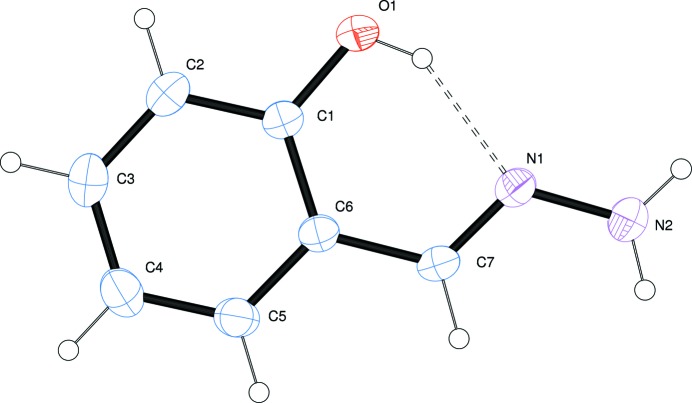
A view of the title compound with displacement ellipsoids drawn at the 30% probability level. Dashed line indicates hydrogen bonding interactions.

**Fig. 2.**
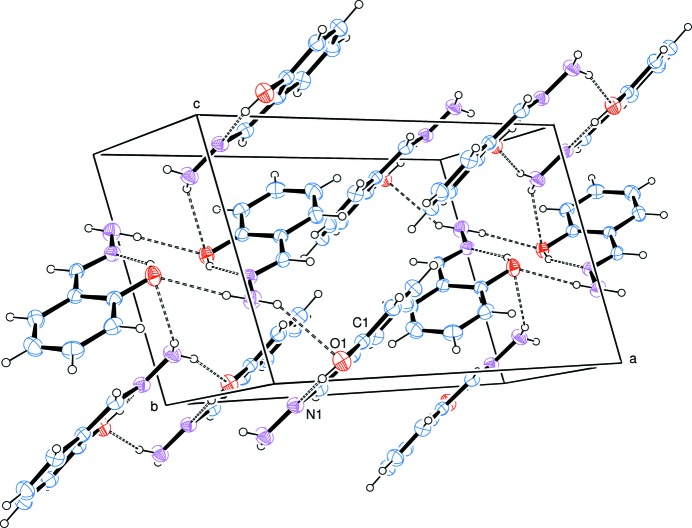
Crystal packing of the title compound.

## supplementary crystallographic information

### Comment

Schiff bases are one of the most prevalent and important mixed-donor ligand in coordination chemistry (Lee *et al.*, 2005). Recently, the synthesis, structure and properties of Schiff base complexes have stimulated much more interest for their noteworthy contributions in pharmaceutical and medicinal activity (Sriram *et al.*, 2006; Hao 2009; Bedia *et al.*, 2006).

The X-ray structural analysis confirmed the assignment of the structure of the title compound(I). The molecular structure is depicted in Fig. 1, and the crystal packing of the title compound(I) is depicted in Fig. 2. In the crystal structure,intermolecular N—H···O, N—H···N and intramolecular O—H···N hydrogen bonds contribute to form the title compound(I).

### Experimental

35% of hydrazine hydrate (0.50 mL, 10 mmol) and salicylidence (0.52 mL, 5 mmol) were mixed in 50.0 mL ethanol and refluxed for 3 h. When the solution was cooled to room temperature, a light yellow solid was obtained, and light yellow block shaped crystals were formed from the filtrate by slow evaporation of the solution in air after a few days. The yield of the isolated yellow solid was 0.62 g.(90%).

### Refinement

H atoms attached to C were placed in geometrically idealized positions with C*sp*^2^—H = 0.93 Å and *U*_iso_(H) = 1.2*U*_eq_(C). H atoms bonded to N and O were located in a difference map. They were refined using a riding model with O—H = 0.82 Å and N—H = 0.86 Å and *U*_iso_(H) = 1.2*U*_eq_(N) or 1.5*U*_eq_(O).

### Crystal data

**Table:**

C_7_H_8_N_2_O	*F*(000) = 288
*M**_r_* = 136.15	*D*_x_ = 1.334 Mg m^−^^3^
Monoclinic, *P*2_1_/*c*	Mo *K*α radiation, λ = 0.71073 Å
Hall symbol: -P 2ybc	Cell parameters from 2298 reflections
*a* = 14.1010 (11) Å	θ = 3.0–28.4°
*b* = 6.0062 (5) Å	µ = 0.09 mm^−^^1^
*c* = 8.1979 (6) Å	*T* = 296 K
β = 102.525 (1)°	Block, yellow
*V* = 677.78 (9) Å^3^	0.46 × 0.45 × 0.35 mm
*Z* = 4	

### Data collection

**Table:**

Bruker SMART CCD area-detector diffractometer	1203 independent reflections
Radiation source: fine-focus sealed tube	1081 reflections with *I* > 2σ(*I*)
graphite	*R*_int_ = 0.013
φ and ω scans	θ_max_ = 25.1°, θ_min_ = 3.0°
Absorption correction: multi-scan (*SADABS*; Bruker, 2000)	*h* = −15→16
*T*_min_ = 0.959, *T*_max_ = 0.968	*k* = −6→7
3351 measured reflections	*l* = −9→8

### Refinement

**Table:**

Refinement on *F*^2^	Secondary atom site location: difference Fourier map
Least-squares matrix: full	Hydrogen site location: inferred from neighbouring sites
*R*[*F*^2^ > 2σ(*F*^2^)] = 0.036	*w* = 1/[σ^2^(*F*_o_^2^) + (0.0587*P*)^2^ + 0.1774*P*] where *P* = (*F*_o_^2^ + 2*F*_c_^2^)/3
*wR*(*F*^2^) = 0.110	(Δ/σ)_max_ < 0.001
*S* = 1.06	Δρ_max_ = 0.33 e Å^−^^3^
1203 reflections	Δρ_min_ = −0.29 e Å^−^^3^
93 parameters	Extinction correction: *SHELXL97* (Sheldrick, 2008), Fc^*^=kFc[1+0.001xFc^2^λ^3^/sin(2θ)]^-1/4^
0 restraints	Extinction coefficient: 0.129 (12)
Primary atom site location: structure-invariant direct methods	

### Special details

**Table:**

Geometry. All e.s.d.'s (except the e.s.d. in the dihedral angle between two l.s. planes) are estimated using the full covariance matrix. The cell e.s.d.'s are taken into account individually in the estimation of e.s.d.'s in distances, angles and torsion angles; correlations between e.s.d.'s in cell parameters are only used when they are defined by crystal symmetry. An approximate (isotropic) treatment of cell e.s.d.'s is used for estimating e.s.d.'s involving l.s. planes.
Refinement. Refinement of *F*^2^ against ALL reflections. The weighted *R*-factor *wR* and goodness of fit *S* are based on *F*^2^, conventional *R*-factors *R* are based on *F*, with *F* set to zero for negative *F*^2^. The threshold expression of *F*^2^ > σ(*F*^2^) is used only for calculating *R*-factors(gt) *etc*. and is not relevant to the choice of reflections for refinement. *R*-factors based on *F*^2^ are statistically about twice as large as those based on *F*, and *R*- factors based on ALL data will be even larger.

### Fractional atomic coordinates and isotropic or equivalent isotropic displacement parameters (Å^2^)

**Table:**

	*x*	*y*	*z*	*U*_iso_*/*U*_eq_	
C1	0.22022 (10)	−0.1162 (2)	0.13139 (16)	0.0374 (4)	
C2	0.27892 (11)	−0.2576 (3)	0.24310 (18)	0.0462 (4)	
H2	0.2554	−0.3958	0.2670	0.055*	
C3	0.37226 (12)	−0.1944 (3)	0.3192 (2)	0.0546 (5)	
H3	0.4111	−0.2898	0.3948	0.065*	
C4	0.40836 (11)	0.0097 (3)	0.2837 (2)	0.0568 (5)	
H4	0.4715	0.0513	0.3339	0.068*	
C5	0.34970 (11)	0.1512 (3)	0.17277 (19)	0.0487 (4)	
H5	0.3743	0.2881	0.1487	0.058*	
C6	0.25465 (9)	0.0945 (2)	0.09582 (16)	0.0372 (4)	
C7	0.19290 (10)	0.2536 (2)	−0.01248 (16)	0.0392 (4)	
H7	0.2190	0.3883	−0.0376	0.047*	
N1	0.10354 (8)	0.21085 (19)	−0.07344 (14)	0.0407 (3)	
N2	0.04759 (9)	0.3659 (2)	−0.17427 (15)	0.0507 (4)	
H2A	0.0693	0.4889	−0.2059	0.061*	
H2B	−0.0089	0.3460	−0.1530	0.061*	
O1	0.12928 (7)	−0.18636 (16)	0.05811 (13)	0.0476 (3)	
H1	0.0993	−0.0840	0.0039	0.071*	

### Atomic displacement parameters (Å^2^)

**Table:**

	*U*^11^	*U*^22^	*U*^33^	*U*^12^	*U*^13^	*U*^23^
C1	0.0428 (8)	0.0355 (7)	0.0358 (7)	−0.0005 (6)	0.0127 (6)	−0.0036 (5)
C2	0.0587 (9)	0.0381 (8)	0.0439 (8)	0.0040 (6)	0.0157 (7)	0.0026 (6)
C3	0.0576 (10)	0.0566 (10)	0.0467 (9)	0.0148 (8)	0.0050 (7)	0.0038 (7)
C4	0.0439 (8)	0.0645 (11)	0.0574 (10)	0.0009 (8)	0.0007 (7)	−0.0041 (8)
C5	0.0468 (8)	0.0447 (8)	0.0543 (9)	−0.0070 (6)	0.0106 (7)	−0.0034 (7)
C6	0.0418 (7)	0.0351 (7)	0.0363 (7)	−0.0016 (6)	0.0118 (5)	−0.0038 (5)
C7	0.0471 (8)	0.0323 (7)	0.0399 (7)	−0.0060 (6)	0.0130 (6)	0.0003 (6)
N1	0.0468 (7)	0.0364 (6)	0.0385 (6)	−0.0013 (5)	0.0083 (5)	0.0020 (5)
N2	0.0523 (8)	0.0466 (8)	0.0522 (8)	0.0037 (6)	0.0092 (6)	0.0140 (6)
O1	0.0451 (6)	0.0353 (6)	0.0607 (7)	−0.0054 (4)	0.0078 (5)	0.0039 (5)

### Geometric parameters (Å, °)

**Table:**

C1—O1	1.3597 (16)	C5—C6	1.3941 (19)
C1—C2	1.384 (2)	C5—H5	0.9300
C1—C6	1.409 (2)	C6—C7	1.4574 (19)
C2—C3	1.382 (2)	C7—N1	1.2768 (18)
C2—H2	0.9300	C7—H7	0.9300
C3—C4	1.382 (2)	N1—N2	1.3749 (16)
C3—H3	0.9300	N2—H2A	0.8604
C4—C5	1.381 (2)	N2—H2B	0.8604
C4—H4	0.9300	O1—H1	0.8200
			
O1—C1—C2	118.26 (12)	C4—C5—H5	119.1
O1—C1—C6	121.42 (12)	C6—C5—H5	119.1
C2—C1—C6	120.32 (13)	C5—C6—C1	117.79 (13)
C3—C2—C1	120.28 (14)	C5—C6—C7	120.29 (13)
C3—C2—H2	119.9	C1—C6—C7	121.88 (12)
C1—C2—H2	119.9	N1—C7—C6	121.00 (12)
C2—C3—C4	120.46 (15)	N1—C7—H7	119.5
C2—C3—H3	119.8	C6—C7—H7	119.5
C4—C3—H3	119.8	C7—N1—N2	119.21 (12)
C5—C4—C3	119.27 (15)	N1—N2—H2A	124.6
C5—C4—H4	120.4	N1—N2—H2B	102.7
C3—C4—H4	120.4	H2A—N2—H2B	125.9
C4—C5—C6	121.86 (14)	C1—O1—H1	109.5
			
O1—C1—C2—C3	179.29 (13)	O1—C1—C6—C5	−178.27 (12)
C6—C1—C2—C3	−0.8 (2)	C2—C1—C6—C5	1.82 (19)
C1—C2—C3—C4	−0.5 (2)	O1—C1—C6—C7	4.02 (19)
C2—C3—C4—C5	0.8 (2)	C2—C1—C6—C7	−175.89 (12)
C3—C4—C5—C6	0.3 (2)	C5—C6—C7—N1	−174.54 (13)
C4—C5—C6—C1	−1.6 (2)	C1—C6—C7—N1	3.1 (2)
C4—C5—C6—C7	176.17 (13)	C6—C7—N1—N2	179.61 (11)

### Hydrogen-bond geometry (Å, °)

**Table:**

*D*—H···*A*	*D*—H	H···*A*	*D*···*A*	*D*—H···*A*
N2—H2A···O1^i^	0.86	2.56	3.3076 (17)	145
N2—H2B···O1^ii^	0.86	2.23	3.0530 (16)	160
O1—H1···N1	0.82	1.89	2.6109 (15)	147

Symmetry codes: (i) *x*, −*y*+1/2, *z*−1/2; (ii) −*x*, −*y*, −*z*.
